# Frontal Sinusitis with Mixed Bacterial Colonies Treated with the Combination of Endoscopic Modified Lothrop Procedure and External Approach

**DOI:** 10.1155/2013/541843

**Published:** 2013-12-09

**Authors:** Kazuhiro Nomura, Yohei Honkura, Yuri Okumura, Atsuko Kasajima, Takahiro Suzuki, Toshiaki Kikuchi, Hiroshi Hidaka, Takeshi Oshima, Yukio Katori

**Affiliations:** ^1^Department of Otolaryngology-Head and Neck Surgery, Tohoku University Graduate School of Medicine, 1-1 Seiryo-cho, Aoba-ku, Sendai, Miyagi 980-8574, Japan; ^2^Department of Pathology, Tohoku University Hospital, 1-1 Seiryo-cho, Aoba-ku, Sendai, Miyagi 980-8574, Japan

## Abstract

Isolated frontal sinusitis with mixed bacterial colonies is extremely rare and has not been described. We report a case of isolated frontal sinus forming mixed bacterial colonies that occurred in the previously exposed frontal sinus. The material in the frontal sinus was macroscopically similar to sinus fungus ball. Surgical strategy followed that for sinus fungus ball. The material could not be completely removed even with an endoscopic modified Lothrop procedure (Draf type III procedure). Additional external incision enabled complete removal of the remnant infectious substance. Histological examination detected two different types of organisms as intermingled bacterial colonies. External approaches to the frontal fungus ball have recently been replaced by the endonasal approach. Our case suggests that material trapped in a pit or small crevice in a frontal sinus may not be removed intranasally.

## 1. Introduction

Most cases of frontal sinusitis are caused by drainage congestion as a consequence of the complex anatomy of the frontal recess, but the infection may also spread via normal anatomical fissures or fracture lines [1]. The standard treatment technique for chronic frontal sinusitis is endoscopic removal of the uncinate process, ethmoid bulla, and common wall between the frontal sinus, the agger nasi cell, and supraorbital cell. Failure to achieve adequate removal of these walls may result in chronic edema and frontal sinus obstruction may develop [2]. The contents of the frontal sinus are mucous, pus, or mucin and can be removed with malleable suction or irrigation.

Sinus fungus ball is a form of fungal sinusitis which is defined as a noninvasive chronic fungal sinusitis without inspissated allergic mucin. Sinus fungus ball occurs in immunocompetent hosts and endoscopic surgical treatment usually results in good outcome. Sinus fungus ball occurs most commonly in the maxillary or sphenoid sinuses [3, 4]. The standard treatment of fungus ball is complete removal of the fungus and wide opening of the ostium of the diseased sinus. Fungus ball of the frontal sinus is extremely rare, with fewer than 40 cases reported in the English literature [5, 6]. The surgical procedure is more difficult than those used in the maxillary and sphenoid sinuses because the possible maximum opening in the case of frontal sinus is relatively small [7]. Previously, almost all cases were treated with an external approach [5]. Recently, with the development of new instruments and innovations in endoscopic techniques, the Draf type III/endoscopic modified Lothrop procedure (EMLP) has been widely used to treat intractable frontal sinus disease [8]. Therefore, the entire frontal sinus fungus ball could be treated with the endonasal approach.

Here, we describe an interesting case of isolated frontal sinusitis with mixed bacterial colonies resembling fungus ball that could not be completely removed with the EMLP, so a portion of the material located in the bony fissure of the frontal sinus was completely removed through an additional external approach. Our case suggests the uses and limits of the EMLP.

## 2. Case Report

**Figure 1:**
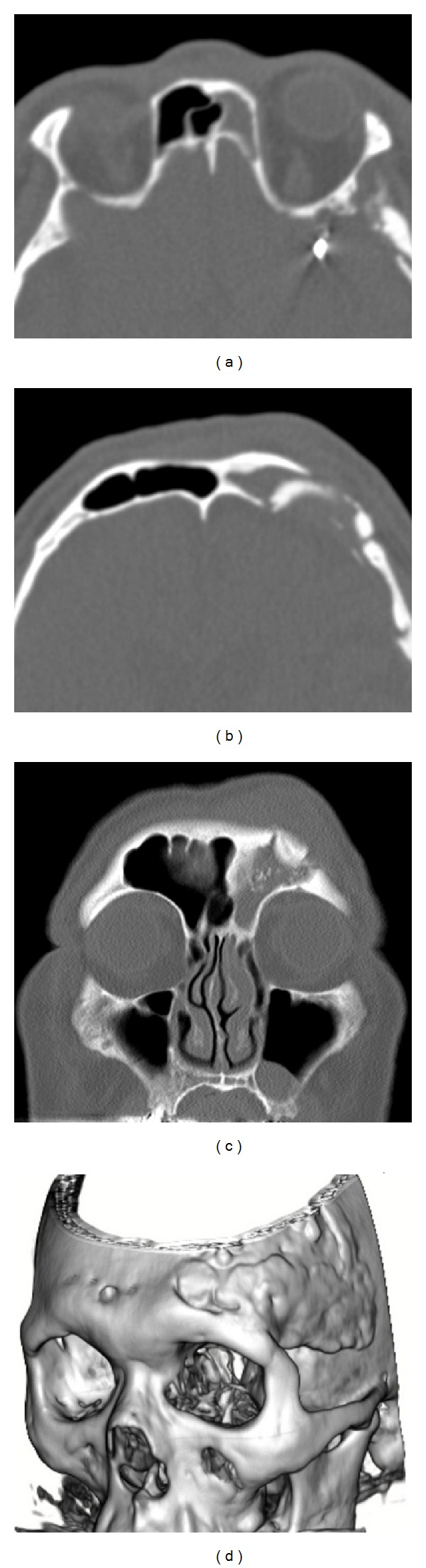
Preoperative computed tomography scans. (a) Axial scan. Left frontal sinus is opacified. Intracranial clip is seen. (b) Axial scan. Frontal bone is deformed as a result of previous neurosurgery. Left frontal sinus has corners with acute angles. (c) Coronal scan. High-density spot is seen in the left frontal sinus. The corners of the frontal sinus are acute. (d) Three-dimensional reconstruction. Postoperative change is seen in the left frontal bone.

A 65-year-old male with repeated episodes of forehead swelling was referred to our department. He had no history of sinus surgery but had undergone microsurgical aneurysm clipping at the age of 47. He did not have diabetes or other immunocompromising disorders. Axial computed tomography demonstrated an intracranial metal-density spot, indicating the site of previous clipping (Figure 1(a)). The posterior wall of the left frontal sinus was deformed as a result of the previous neurosurgery (Figure 1(b)). The left middle meatus was opacified with homogeneous density (Figure 1(c)). The left frontal sinus had heterogeneous opacity including a high-density spot. Three-dimensional reconstruction showed the postoperative change of the frontal bone (Figure 1(d)).

**Figure 2:**
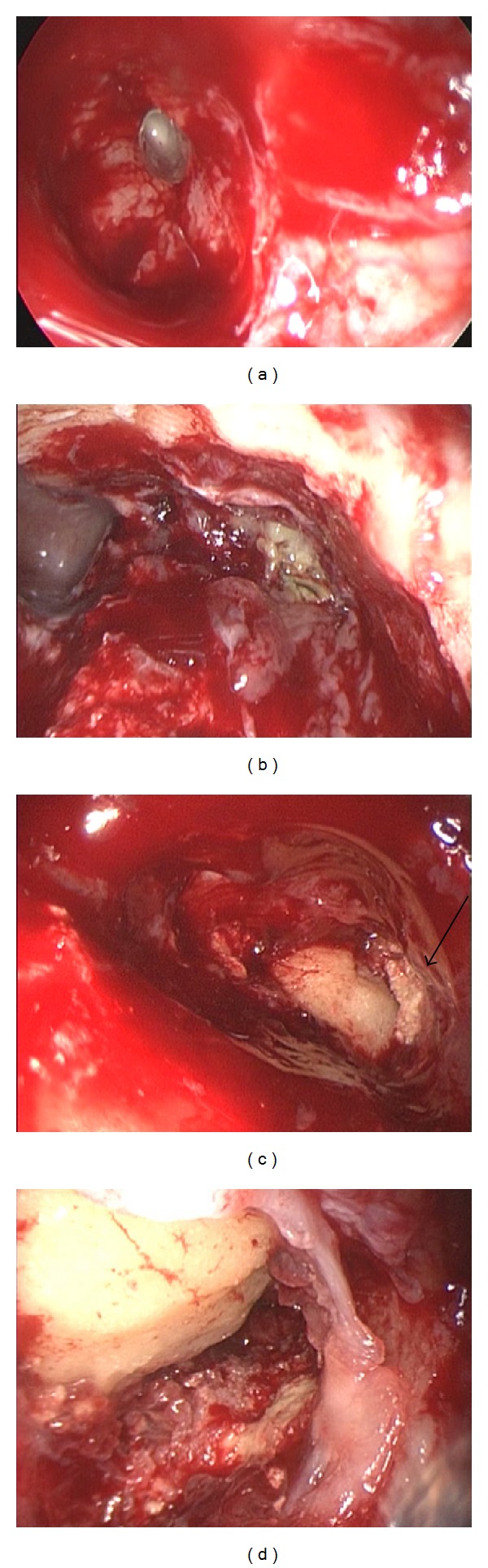
Intraoperative endoscopic view. (a) Frontal sinus opened with conventional approach (Draf type IIa) seen through a 70° endoscope. (b) Frontal sinus opened with the EMLP seen through a 70° endoscope. Fungus ball is located in the left frontal sinus. (c) Left frontal sinus opened with the EMLP seen through a 70° endoscope. Small portion of the fungus ball is located in the fissure (arrow). (d) Corner where the fungus ball was trapped was seen through the external approach.

**Figure 3:**
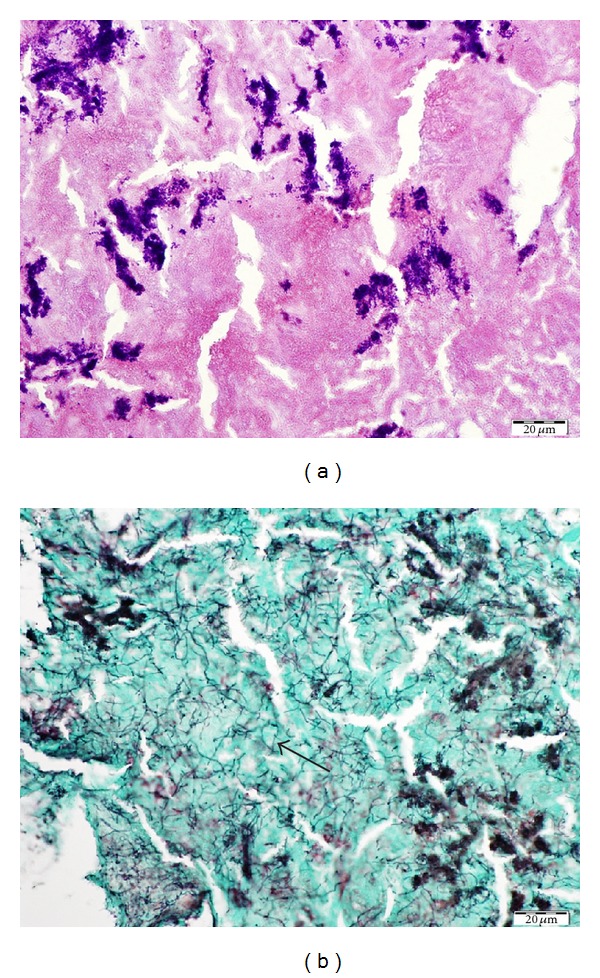
(a) Gram-positive cocci observed by Gram staining. (b) Numerous long filament structures, approximately 2-3 *μ*m in diameter, positive for Grocott staining (arrow).

Surgery was performed under general anesthesia. The mucosa of the frontal recess was edematous and obstructed the drainage pathway of the frontal sinus. Total removal of the anterior ethmoid cells revealed the frontal sinus ostium, but this ostium was too narrow to introduce any instrument into the frontal sinus (Figure 2(a)). Therefore, the EMLP was performed. The bilateral frontal sinuses were opened and the frontal beak and intersinus septum were drilled out. The left frontal sinus was filled with cheesy brown material (Figure 2(b)). Most of the material could be removed with curved instruments and flexible suction devices, but a portion of the material was located in the fissure of the bone. Even meticulous lavage with saline and extensive adjustment of flexible instruments failed to remove all of the remnant material (Figure 2(c)). Therefore, a small skin incision was made at the eyebrow and the frontal wall of the frontal sinus was trephined. Through this direct and close approach, the entire remainder of the material could be removed (Figure 2(d)). We preserved the mucosa of the frontal sinus to prevent postoperative scarring at the surgery site. The nasal cavity was packed with Sorbsan (calcium alginate) [9]. Histological examination revealed that the purulent material was formed by mixed bacterial colonies of Gram-positive coccus and an organism with long filament formation resembling actinomyces (Figure 3). The symptom was resolved after the operation. The frontal sinus ostium was open and no infectious material was apparent at an outpatient visit.

## 3. Discussion

Frontal sinusitis with mixed bacterial colonies is extremely rare and has not been described previously. In the present case, the frontal sinus was filled with cheesy, friable brown material which resembled sinus fungus ball. Preoperative computed tomography had indicated high-density mass in the frontal sinus, which is also typical of sinus fungus ball. The computed tomography appearance and intraoperative findings of cheesy brown material convinced us that the case was frontal sinus fungus ball. Since the definitive diagnosis is based on pathological examination with specific staining, which could not be performed intraoperatively, we adopted the surgical technique used for frontal sinus fungus ball. Frontal sinus fungus ball has previously been treated with conventional external incision, including external fronto-ethmoidectomy, osteoplastic flap, and trephination of the frontal sinus [5]. The endoscopic approach has only recently been advocated to treat frontal sinus fungus ball [5, 6, 10]. Sixteen cases of primary frontal sinus aspergillosis, including fungus ball (4 cases) and allergic fungal rhinosinusitis (12 cases), were treated first by endoscopic sinus surgery, and the recurrent 3 cases were treated by the EMLP [5]. Two other cases were treated with Draf type II frontal sinusotomy or with the external approach via coronal incision because neoplasm was suspected based on a large defect in the posterior wall of the frontal sinus [6]. In another case, the EMLP was performed because of the complex anatomy [10]. In these three series, the frontal sinus fungus ball could be successfully treated using endoscopy without external incision [5, 6, 10].

In our case, simple endoscopic frontal sinusotomy (Draf type IIa) could not access the frontal sinus contents because of the limited anterior-posterior distance and hypertrophic frontal sinus mucosa. Therefore, we performed the EMLP. Most of the fungus ball was removed but a small amount persisted in a crevice at the posterior wall formed as a result of previous neurosurgery. Even saline irrigation with a malleable suction tube and scraping with malleable instruments could not remove a portion of the infectious material in the crevice. Finally, external skin incision at the eyebrow and trephination of the anterior frontal sinus enabled the removal of all the material.

The stepwise approach is preferable to treat frontal sinus fungus ball, as Draf type IIa can be changed to type III intraoperatively. Additional external incision is required if small amounts of residual material persist but can be safely performed using light guides since the frontal sinus has been identified by the EMLP. Flexible decision making is essential when performing complex frontal sinus surgery. Our case suggests that not all such cases can be treated with only the endoscopic approach. Although frontal sinusitis with fungus ball or other infectious materials is rare, similar conditions may occur in the presence of well-pneumatized frontal sinus with intersinus septum. Surgeons should always bear in mind that external incision might be necessary even with the EMLP.

Frontal sinusitis occurs mostly due to the inadequate drainage based on the complex anatomy of the frontal recess, but bacterial infection is not an uncommon cause. The organisms observed in this case were a mixture of two different organisms, colonized Gram-positive cocci (Figure 3(a)) and thin and long filament structures positive for Grocott staining (Figure 3(b)). The latter organism morphologically resembled actinomyces and was not identical to any fungal characteristics, but gram staining did not reveal its structure. Although no bacteria or fungi were detected by the culture test, our final diagnosis was mixed bacterial infections based on the histopathological findings.

In conclusion, most cases of frontal sinusitis with fungal ball or infectious material can be treated with only the intranasal approach. However, materials trapped in a pit or small crevice in a frontal sinus may not be removed intranasally. Additional external incision should be considered in such a case.
